# Role of human urinary kallikrein in reducing progressive ischemic stroke among acute ischemic stroke patients with concurrent hypertension and diabetes: a hospital-based retrospective cohort study

**DOI:** 10.3389/fneur.2025.1520309

**Published:** 2025-06-10

**Authors:** Zeyang Zheng, Yuelong Li, Shanshan Yang, Yuanqi Xu, Lian Yi, Yushuang Liu, Li Zhang, Zhongling Zhang

**Affiliations:** Department of Neurology, The First Affiliated Hospital of Harbin Medical University, Harbin, China

**Keywords:** acute ischemic stroke, progressive ischemic stroke, human urinary kallikrein, stroke management, vascular pathology

## Abstract

**Background:**

Progressive ischemic stroke (PIS) poses significant challenges in the management of acute ischemic stroke (AIS), with higher morbidity and mortality rates, especially among patients with vascular risk factors such as hypertension and diabetes. This study evaluates the efficacy of human urinary kallidinogenase (HUK) in reducing the incidence of PIS in patients with AIS, with a particular focus on subgroups based on vascular pathology and thrombolytic treatment.

**Methods:**

This retrospective cohort study included 916 patients with AIS treated at a single tertiary care center between January 2022 and September 2023. The patients were divided into two groups based on whether they received HUK treatment in addition to standard care or standard care alone. The primary outcome was the incidence of PIS. Independent sample t-tests or chi-squared tests were used for univariate analysis between groups to identify potential predictors associated with the occurrence of PIS, with factors achieving a *p*-value < 0.1 considered for multivariate binary logistic regression analysis. Multivariate analysis adjusted for potential confounders to determine independent predictors significantly associated with PIS. The significance threshold was set at *p* < 0.05. In addition, subgroup analyses were conducted based on stroke subtype (TOAST classification), thrombolysis treatment, and infarction location.

**Results:**

HUK treatment significantly reduced the incidence of PIS (*p* < 0.001), with the most notable effects observed in patients with large-artery atherosclerosis and small-artery occlusion, those not undergoing intravenous thrombolysis, and those with anterior circulation infarctions. Conversely, no significant reduction was noted in patients with cardioembolic stroke, other etiologies of infarction, intravenous thrombolysis, posterior circulation infarctions, or both anterior and posterior circulation infarctions. Factors such as low body mass index (BMI) and high activated partial thromboplastin time are associated with an increased risk of PIS.

**Conclusion:**

HUK treatment appears to be an effective strategy for reducing the risk of PIS in patients with AIS, particularly in those at higher risk owing to specific vascular pathologies. These findings support the use of HUK in clinical practice to improve the outcomes of patients with stroke. Future prospective, multicenter, randomized controlled trials are warranted to validate these findings and further elucidate the underlying mechanisms.

## 1 Introduction

Stroke remains the second leading cause of death worldwide and the third most common cause of disability and mortality. Ischemic strokes, representing 62.4% of all new stroke cases, disproportionately affect populations in low to upper-middle-income countries, accounting for over 80% of stroke-related disability-adjusted life years (DALYs) (1). Notably, approximately 25 to 33% of stroke survivors develop PIS within days following the initial event, presenting with worsening neurological deficits and generally poorer prognostic outcomes (2).

HUK, a serine protease derived from urine, has garnered attention for its therapeutic potential in AIS, primarily through enhancing collateral circulation, stimulating angiogenesis, and improving cerebral perfusion (3–5). A longitudinal study involving 300 patients demonstrated that those receiving HUK exhibited notably lower scores on the modified Rankin Scale (mRS) at a 12-month follow-up compared to their counterparts in the control group (6). Moreover, a meta-analysis incorporating data from 24 studies quantified the neurologic improvement attributable to HUK, indicating a 0.56-fold increase in recovery rates (7). Focusing on patients with large artery atherosclerosis, another retrospective analysis revealed that HUK treatment was associated with significantly reduced National Institutes of Health Stroke Scale (NIHSS) scores (8).

Hypertension and diabetes are prevalent risk factors in the AIS patient demographic, with the National Inpatient Sample highlighting that 79% of these patients suffer from hypertension, and 34% are diabetic (9–11). Endothelial dysfunction, insulin resistance, and impaired vascular reactivity caused by hypertension and diabetes may increase the adverse risks in AIS patients. Previous studies have demonstrated that blood pressure levels in AIS patients are significantly associated with their neurological outcomes. Maintaining appropriate blood pressure levels can significantly improve the prognosis of AIS patients (12, 13). Given the high prevalence of these comorbidities and their significant impact on stroke outcomes, investigating the therapeutic potential in this high-risk subgroup is of great clinical significance. Despite these statistics, research into HUK’s effectiveness specifically for patients with AIS concurrently diagnosed with these conditions remains sparse. One study reported that patients with AIS with stage 3 hypertension undergoing HUK treatment showed substantial improvements in mRS scores and recovery rates 3 months post-treatment (14). Another study contrasting patients with AIS with abnormal glucose metabolism observed a significant reduction in NIHSS scores following HUK treatment, although mRS scores did not differ significantly between the treated and control groups (15).

Thus, the present study aimed to investigate the effects of HUK on the incidence of PIS post-admission in patients with AIS with both hypertension and with diabetes, with a subgroup analysis by site of lesions, TOAST subtypes, and the use of intravenous thrombolysis.

## 2 Methods

### 2.1 Study design and participants

This retrospective cohort study included 916 patients who presented with mild (NIHSS ≤ 7) or moderate (NIHSS: 8–16) AIS and were admitted to the Department of Neurology at the First Hospital of Harbin Medical University between January 2022 and September 2023 (16). Eligible participants had a confirmed diagnosis of hypertension and diabetes either prior to or during their current treatment.

The study protocol was approved by the Institutional Review Board of the First Hospital of Harbin Medical University (IRB-2023326) and adhered to the ethical standards of the Declaration of Helsinki. All participants provided written informed consent.

Patients were stratified into two groups based on their treatment regimens. The experimental group received HUK treatment (0.15 PNA units/day intravenously) alongside standard care, which included antiplatelet aggregation therapy, lipid-lowering drugs, potential intravenous thrombolysis, and antifibrinolytic treatment for 6 ± 1 days. The control group received only the standard care regimen.

PIS was defined as an increase in the NIHSS score by two or more points from baseline within 6 h to 7 days post-onset of the initial cerebral infarction (17). In diagnosing PIS, patients with alternative causes of neurological deterioration, such as hemorrhagic transformation, recurrent embolism, systemic infections, or hypotension, were excluded. Nevertheless, it is possible that other potential causes of deterioration, such as seizures or metabolic disturbances, were not entirely excluded in this retrospective study.

### 2.2 Subgroup definitions

Patients were further categorized into thrombolytic and non-thrombolytic subgroups based on their receipt of intravenous thrombolytic agents (e.g., alteplase, urokinase). Additionally, according to the Trial of Org 10,172 in Acute Stroke Treatment (TOAST) classification (18), the causes of AIS are subdivided into large artery atherosclerosis, cardioembolic disease, small vessel occlusion, other determined causes, and undetermined causes. The location of the cerebral lesions was classified as either an anterior or a posterior circulation infarction.

### 2.3 Inclusion and exclusion criteria

Participants were required to meet several criteria to ensure a uniform study population. Each patient was diagnosed with AIS according to the 2018 Chinese Guidelines for the Diagnosis and Treatment of Acute Ischemic Stroke (19). Additionally, all participants had a concurrent diagnosis of hypertension and diabetes as outlined in the 2022 Chinese Hypertension Clinical Practice Guidelines and 2023 American Diabetes Diagnosis and Treatment Standards. Eligible patients either experienced their first clinical episode or had a history of cerebral infarction without severe sequelae, as indicated by a mRS score of 0–2. Age criteria were set for participants aged from 18 to 80 years. Further requirements included symptom onset within 3 days prior to hospital admission and a completed hospital stay of approximately 6 ± 1 days during which comprehensive clinical data were collected.

Exclusion criteria were as follows. Certain conditions and scenarios excluded potential participants from the study to control confounding variables and focus on the target population. Patients who underwent mechanical thrombectomy after stroke onset were excluded. Patients with severe complications related to hypertension or diabetes, such as malignant hypertension or diabetic ketoacidosis, which could influence study outcomes, were also excluded, as well as patients presenting with large-area cerebral infarction, coma, or brain herniation on admission. Additionally, significant co-morbid conditions, such as severe cardiovascular, hematological, respiratory, or hepatic-renal impairments, were excluded from the study to maintain participant safety and data integrity.

**Figure 1:**
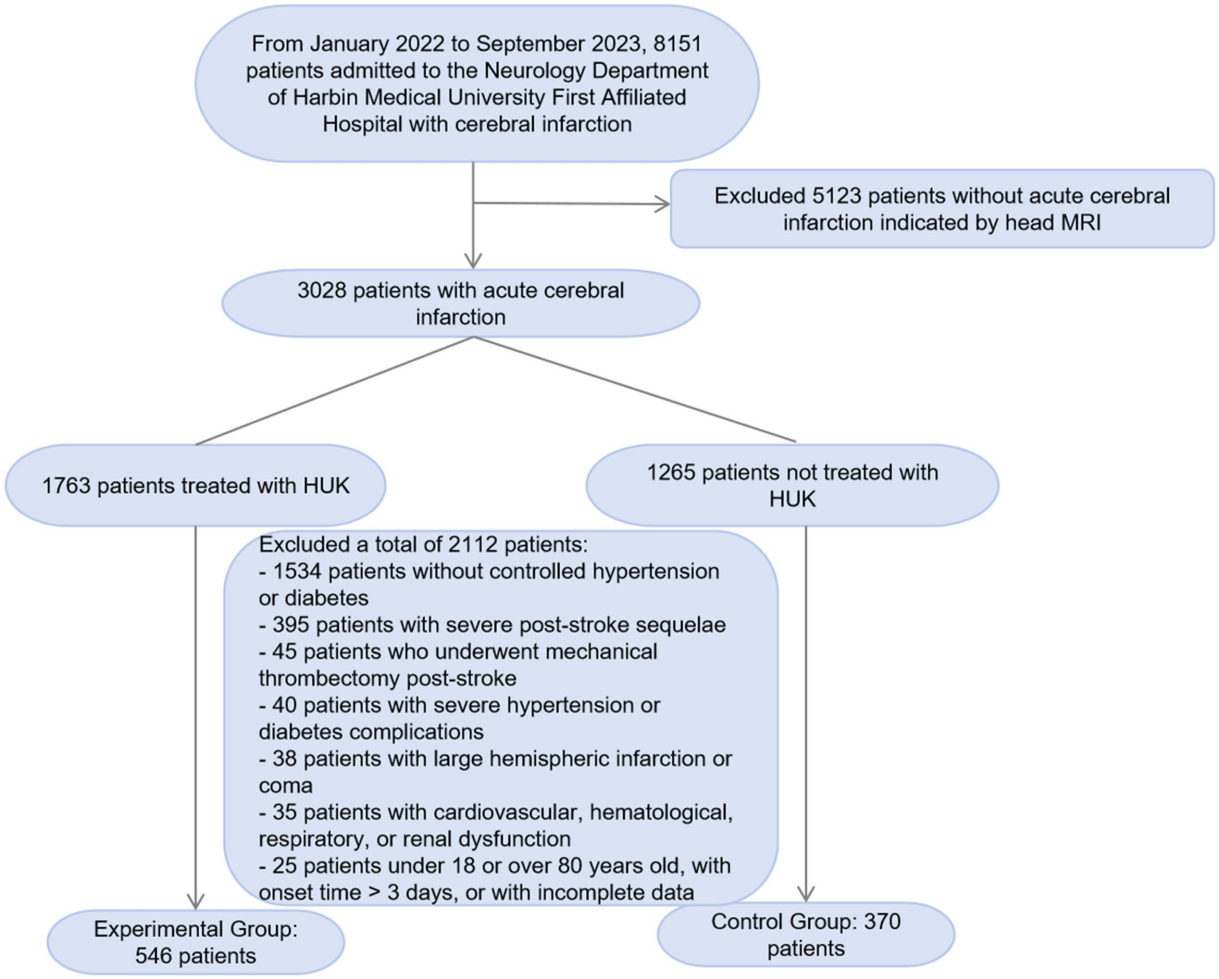
The flow chat of the patients’ collection.

In this study, we screened 8,151 patients with cerebral infarction admitted to the Neurology Department of the First Affiliated Hospital of Harbin Medical University from January 2022 to September 2023. We excluded 5,123 patients whose head MRI did not indicate acute cerebral infarction. Among the remaining 3,028 patients with acute cerebral infarction, we further excluded 2,112 patients who did not meet the study criteria. The exclusion criteria included 1,534 patients without a typical history of hypertension and diabetes, 395 patients with a history of cerebral infarction and severe sequelae, 45 patients who underwent mechanical thrombectomy after the onset of the disease, 40 patients with severe hypertension or diabetes complications, 38 patients with large hemispheric infarction, coma, and cerebral herniation, and 35 patients with severe diseases such as cardiovascular, hematological, respiratory, and renal dysfunction. Additionally, we excluded 25 patients who were outside the age range of 18–80 years, had an onset time exceeding 3 days, or had incomplete data. Ultimately, we included 546 patients in the experimental group and 370 patients in the control group. We have added a flowchart (Figure 1) to the manuscript to illustrate the screening and exclusion process of the participants in detail.

### 2.4 Collected data and quality control

Personal and clinical data collected included demographics, medical history (e.g., stroke, cerebral hemorrhage, coronary artery disease, and arrhythmias), lifestyle factors (smoking and alcohol consumption), and admission vitals (blood pressure, heart rate, and body temperature). Laboratory data included complete blood count, coagulation profile, fasting glucose level, lipid profile, liver and renal function tests, and other relevant biochemical markers. Imaging assessments included MRI, magnetic resonance angiography, diffusion-weighted imaging, computed tomography angiography, and digital subtraction angiography of head and neck vessels.

The data for this study were sourced from the electronic medical record (EMR) system of the First Hospital of Harbin Medical University, which captures comprehensive clinical information of patients, including demographic data, medical history, laboratory results, imaging findings, and treatment details. To ensure data quality, a multi-step validation process was implemented. Data entry was performed by trained medical staff and verified by a second independent reviewer. Any discrepancies were resolved through consensus discussions. Additionally, a data cleaning process was conducted to identify and correct inconsistencies or missing values. This rigorous approach ensured the accuracy and completeness of the dataset used for analysis.

### 2.5 Statistical analysis

Continuous variables following a normal distribution were expressed as mean ± standard deviation and analyzed using independent sample t-tests. Categorical variables were presented as frequencies and percentages and analyzed using chi-squared tests. Univariate analysis was conducted to identify potential predictors associated with the occurrence of progressive ischemic stroke (PIS). Factors with a *p*-value < 0.1 in the univariate analysis were considered to have potential significance and were included in the multivariate binary logistic regression analysis. The multivariate analysis adjusted for potential confounders, including sex, age, body mass index (BMI), history of coronary heart disease, alcohol consumption, stroke location, HUK treatment, and other relevant factors, to determine the independent predictors significantly associated with PIS. The significance threshold was set at *p* < 0.05. In addition, subgroup analyses were performed to assess the impact of HUK treatment on different patient subgroups. The subgroups were defined based on the following criteria: (1) TOAST classification, including large-artery atherosclerosis, small-artery occlusion, cardioembolic stroke, etc.; (2) thrombolysis treatment, with patients divided into those who received intravenous thrombolysis and those who did not; and (3) infarction location, with patients divided into those with anterior circulation infarction and those with posterior circulation infarction. In the subgroup analyses, both univariate and multivariate analyses were conducted for each subgroup to evaluate the effect of HUK treatment on the incidence of PIS. For each subgroup, odds ratios (OR) and their 95% confidence intervals (CI) were calculated, and logistic regression models were used for significance testing. Significance levels in the tables and figures are indicated by symbols as follows: + for *p* < 0.1, ∗ for *p* < 0.05, ∗∗ for *p* < 0.01, and ∗∗∗ for *p* < 0.001. Data analysis was conducted using SPSS software version 25.0.

## 3 Results

This study included 916 patients diagnosed with mild and moderate AIS, of whom 546 received HUK treatment, and the remaining 370 served as the control group and received standard treatment only. Overall, 139 (15.2%) patients developed PIS during the study period. Most participants were male (63.1%), with an average age of 63.50 years, illustrating a typical demographic profile for AIS (Table 1). The average NIHSS score upon patient admission was 3.21 points, with males scoring 3.25 points and females scoring 3.14 points. Among them, there were 875 patients (95.5%) with mild stroke and 41 patients (4.5%) with moderate stroke. Among males, there were 550 patients (95.1%) with mild stroke and 28 patients (4.9%) with moderate stroke. Among females, there were 325 patients (95.8%) with mild stroke and 13 patients (3.8%) with moderate stroke.

**Table 1:** Characteristics of participants.

Characteristics	Men	Women	Total
Case, *n* (%)	578 (63.1)	338 (36.9)	916 (100)
Age, means±SD	61.86 ± 9.04	66.31 ± 8.21	63.50 ± 9.00
BMI, means±SD	25.76 ± 3.26	25.63 ± 3.91	25.71 ± 3.51
History of cerebral infarction, *n* (%)	223 (38.6)	130 (38.5)	353 (38.5)
History of cerebral hemorrhage, *n* (%)	26 (4.5)	18 (5.3)	44 (4.8)
Coronary artery disease, *n* (%)	73 (12.6)	63 (18.6)	136 (14.8)
AF and Arrhythmia, *n* (%)	22 (3.8)	13 (3.8)	35 (3.8)
History of anticoagulants, *n* (%)	7 (1.2)	2 (0.6)	9 (1.0)
History of antiplate drugs, *n* (%)	26 (4.5)	10 (3.0)	36 (3.9)
Smoking, *n* (%)	236 (40.8)	34 (10.1)	270 (29.5)
Drinking alcohol, *n* (%)	192 (33.2)	6 (1.8)	198 (21.6)
Stroke location, *n* (%)			
Anterior circulation lesions	302 (52.8)	196 (59.8)	498 (55.3)
Posterior circulation stroke	234 (40.9)	113 (34.5)	347 (38.6)
Anterior+Posterior circulation stroke	36 (6.3)	19 (5.8)	55 (6.1)
Intravenous thrombolysis	49 (8.5)	31 (9.2)	80 (8.7)
TOAST, *n* (%)			
Atherosclerotic type	265 (46.6)	157 (48.2)	422 (47.2)
Cardiogenic embolic type	20 (3.5)	9 (2.8)	29 (3.2)
Small artery occlusion type	204 (35.9)	125 (38.3)	329 (36.8)
Other etiologic determinants	4 (0.7)	0 (0.0)	4 (0.4)
Unexplained type	76 (13.4)	35 (10.7)	111 (12.4)
HUK, *n* (%)	354 (61.2)	192 (56.8)	546 (59.6)
PIS, *n* (%)	78 (13.5)	61 (18.0)	139 (15.2)
Baseline NIHSS	3.25 (2.32)	3.14 (2.15)	3.21 (2.26)
NIHSS group			
Mild	550 (95.1)	325 (96.2)	875 (95.5)
Moderate	28 (4.9)	13 (3.8)	41 (4.5)

### 3.1 Association of HUK treatment with PIS

Univariate analyses highlighted significant differences in stroke location and outcomes between the PIS and non-PIS groups. Specifically, the PIS group had higher baseline NIHSS scores at admission compared to the non-PIS group (*p* < 0.05). The rate of HUK treatment was significantly lower in the PIS group than in the non-PIS group (*p* < 0.001; Supplementary Table 1). Additionally, lipid profile analyses indicated that lipoprotein-associated Lpa levels were significantly elevated in the PIS group (*p* < 0.05; Supplementary Table 2).

Multivariate adjustment for factors including sex, BMI, coronary heart disease, alcohol consumption, stroke location, HUK treatment, and lipid levels confirmed a robust negative correlation between HUK treatment and the occurrence of PIS, with an odds ratio (OR) of 0.33 (95% confidence interval [CI]: 0.22–0.50, *p* < 0.001), suggesting that HUK treatment reduced the incidence of PIS by 67%. Additionally, the analysis revealed that coronary heart disease was associated with a 71% increased risk of PIS (OR = 1.71, 95% CI: 1.03–2.85, *p* = 0.039). Furthermore, each one-unit increase in baseline NIHSS score at admission was associated with a 14% increased risk of PIS (OR = 1.14, 95% CI: 1.04–1.24, *p* = 0.003) (Table 2).

**Table 2:** Results of multifactorial analysis between PIS and non-PIS groups.

Characteristics	References	OR (95% CI)	*p*
HUK	No	0.33 (0.22, 0.50)	<0.001^***^
Male	Women	0.98 (0.63, 1.53)	0.940
BMI	-	0.95 (0.89, 1.00)	0.052^+^
Coronary artery disease	No	1.71 (1.03, 2.85)	0.039^*^
Drinking alcohol	No	0.64 (0.36, 1.15)	0.134
Stroke location	Anterior+Posterior circulation		
Anterior circulation		1.48 (0.65, 3.35)	0.354
Posterior circulation		0.59 (0.25, 1.42)	0.240
TC	—	1.08 (0.81, 1.45)	0.584
ApoB	—	0.95 (0.27, 3.28)	0.933
ApoA /ApoB	—	0.71 (0.40, 1.25)	0.234
Lpa	—	1.00 (1.00, 1.00)	0.384
Baseline NIHSS		1.14 (1.04, 1.24)	0.003^**^

+*p*<0.1, * *p* < 0.05, ** *p* < 0.01, *** *p* < 0.001.

The binary logistic regression model was adjusted for potential confounders, which included factors with a *p*-value < 0.1 in the univariate analysis.

### 3.2 Association of HUK treatment with PIS by TOAST classification

Subgroup analyses revealed variability in stroke outcomes influenced based on the underlying pathologies. Patients with large-artery atherosclerosis who received HUK treatment exhibited a 66% reduction in PIS occurrence (OR = 0.34, 95% CI: 0.19–0.62, *p* < 0.001). Conversely, in the cardioembolic subgroup, elevated blood sodium levels significantly increased the risk of developing PIS (*p* < 0.05). For patients with small artery occlusion, significant disparities were observed in CR, and administration of HUK treatment, with patients with PIS showing lower rates of these factors (all *p* < 0.05; Table 3).

**Table 3:** Association of HUK treatment with PIS in Subgroup analysis.

Subgroups	Unadjusted		Adjusted	
	OR (95% CI)	*p*	OR (95% CI)	*p*
TOAST classification				
Atherosclerotic type	0.39 (0.23, 0.67)	<0.001^***^	0.34 (0.19, 0.62)	<0.001^***^
Cardiogenic embolic type	0.32 (0.05, 2.13)	0.240	-	
Small artery occlusion type	0.41 (0.22, 0.75)	0.004^**^	0.33 (0.17, 0.66)	0.002^**^
Unexplained type	0.26 (0.07, 0.94)	0.040^*^	0.11 (0.001, 13.02)	0.363
Intravenous thrombolysis				
Yes	0.53 (0.18, 1.61)	0.264	-	
No	0.35 (0.24, 0.52)	<0.001^***^	0.33 (0.22, 0.50)	<0.001^***^
Location				
Anterior circulation	0.20 (0.12, 0.32)	<0.001^***^	0.20 (0.12, 0.34)	<0.001^***^
Posterior circulation	1.75 (0.78, 3.93)	0.173	-	
Anterior+Posterior circulation stroke	0.22 (0.04, 1.18)	0.076^+^	-	

+*p*<0.1, * *p* < 0.05, ** *p* < 0.01, *** *p* < 0.001.

The binary logistic regression model was performed both unadjusted and adjusted for potential confounders, which included factors with a *p*-value < 0.1 in the univariate analysis. Univariate analyses of HUK and PIS in the cardioembolic subgroups, intravenous thrombolysis, posterior circulation, and anterior–posterior circulation stroke were not statistically significant. The other pathogen determinants subgroup were due to small sample sizes (*n* = 4). Therefore, no multivariate analysis was performed. The factors adjusted in the multivariate analysis for each subgroup, as well as the detailed results, are presented in Supplementary Table 3.

### 3.3 Association of HUK treatment with PIS by thrombolysis

Differences based on thrombolytic treatment showed that in patients who underwent thrombolysis, smoking prevalence and higher admission body temperatures were more common in the PIS group than in those who did not undergo PIS (*p* all < 0.05). Multivariate analysis showed that a higher body temperature at admission was associated with an increased risk of developing PIS (*p* < 0.05). In contrast, among patients who did not receive intravenous thrombolysis, those in the PIS group were less likely to have received HUK treatment than those in the non-PIS group (*p* < 0.001), and subsequent analyses demonstrated a significant protective effect of HUK against PIS (OR = 0.33, 95% CI: 0.22–0.50, *p* < 0.001; Table 3).

### 3.4 Association between HUK treatment and PIS by infarction location

Analysis revealed that patients with anterior circulation stroke who received HUK treatment experienced a substantial reduction in PIS rates (80% reduction, OR = 0.20, 95% CI: 0.12–0.34, *p* < 0.001). In the posterior circulation subgroup, factors such as the presence of coronary heart disease (OR = 2.61, 95% CI: 1.11–6.17, *p* = 0.028) and antiplatelet drug treatment (OR = 5.29, 95% CI: 1.46–19.19, *p* = 0.011) were more prevalent among those who developed PIS, with significant associations found in the multivariate analysis (*p* < 0.05; Table 3; Supplementary Table 3).

## 4 Discussion

This study retrospectively examined clinical cases to explore the relationship between HUK and the occurrence of PIS in patients with mild to moderate AIS with hypertension and diabetes. The findings revealed that after adjusting for multiple factors, HUK significantly reduced the risk of PIS in patients with AIS with both hypertension and diabetes. Furthermore, low BMI, elevated blood sodium, and elevated activated partial thromboplastin time were identified as risk factors for increased incidence of PIS in patients with AIS. Subgroup analysis further indicated that HUK notably decreased the incidence of PIS in patients with large-artery atherosclerosis, those with small-artery occlusion, those not receiving intravenous thrombolysis, and those with anterior circulation infarction. Conversely, no statistically significant association was found between HUK and PIS in patients with cardioembolic infarctions, those with other etiologies of infarction, those receiving intravenous thrombolysis, those with posterior circulation infarctions, or those with both anterior and posterior circulation infarctions. The differential effects observed may be partially explained by anatomical and physiological factors. For example, large-artery atherosclerosis and anterior circulation strokes may have better collateral flow and greater vascular responsiveness to HUK’s vasodilatory and angiogenic properties, while posterior circulation and cardioembolic strokes may present less opportunity for perfusion enhancement.

Progressive neurological deterioration is the primary characteristic of PIS (20). Studies indicate that the occurrence of PIS is associated with pathophysiological processes, such as insufficient cerebral blood flow perfusion, thrombosis, and ischemic inflammatory responses (2, 20–22). Events of insufficient cerebral blood perfusion, such as thrombus expansion, poor collateral circulation, and blood pressure decline after AIS, can lead to the enlargement of previously ischemic areas. This results in symptomatic ischemic tissue extending to surrounding asymptomatic ischemic regions (penumbra), thereby causing progressive neurological decline (22–26).

Compared to patients with normal blood pressure, stroke patients with hypertension have smaller salvageable tissue (penumbra) and a larger infarct area, leading to poorer stroke outcomes (27–29). Angiotensin II (Ang II), a key hormone of the renin-angiotensin system (RAS), plays a crucial role in the pathophysiology of hypertension. Ang II increases oxidative stress by activating angiotensin II type 1 receptor, which increases the activity of NADPH oxidase, leading to increased vascular tension and endothelial dysfunction (30–32). During hypertension, increased levels of Ang II result in inward remodeling and increased constriction of cerebral arteries and arterioles, increasing cerebrovascular resistance and exacerbating cerebral blood flow insufficiency during AIS. This further leads to a reduced number of ischemic penumbrae, impaired collateral circulation, and an enlarged infarct area (27, 33, 34).

Hyperglycemia-related superoxides, vasoconstrictive endothelin-1, and matrix metalloproteinases contribute to vascular myogenic responses and remodeling (35). Additionally, diabetes-induced changes such as increased collagen deposition, increased vascular stiffness, and endothelial dysfunction lead to increased ischemic injury-related complications and impaired angiogenesis post-stroke (36, 37).

The relationship between HUK and the reduction in PIS in patients with AIS, particularly in those with hypertension or diabetes, has been reported in previous studies. Several studies have explored the effects of HUK in various subgroups of patients with AIS. Wu et al. investigated the effects of HUK in patients with AIS and grade 3 hypertension and found that the HUK treatment group exhibited significantly lower mRS scores after 3 months than the control group, indicating better recovery outcomes (14). Similarly, Chen et al. studied patients with AIS with abnormal glucose metabolism and reported that HUK significantly reduced the NIH Stroke Scale scores in these patients. However, there was no significant difference in the mRS score reduction between the HUK-treated and control group (15). However, our study uniquely focused on patients with AIS with both hypertension and diabetes comorbidities, a high-risk subgroup that has not been thoroughly studied. In comparison with these studies, our findings also indicate that HUK significantly reduced the incidence of PIS in patients with AIS with both hypertension and diabetes, suggesting that HUK treatment reduces the incidence of PIS by 67%.

Few studies have examined the relationship between the HUK and PIS in patients with different types of AIS. Li et al. conducted a study on the efficacy of HUK in patients with AIS classified according to the TOAST criteria and found that HUK treatment improved the outcomes in patients with large-artery atherosclerosis and small-artery occlusion (38). The RESK study demonstrated that HUK is particularly effective in patients with AIS and anterior circulation infarctions (5). Another study involving 92 patients suggested that a combination of HUK and intravenous thrombolysis enhanced neurological function in patients (39). Our study provides a comprehensive evaluation of the effects of HUK on the incidence of PIS across various AIS subtypes. Specifically, we confirmed the effectiveness of HUK in patients with large-artery atherosclerosis, small-artery occlusion, those who did not receive intravenous thrombolysis, and those with anterior circulation infarctions. Our results indicated that HUK significantly decreased the incidence of PIS in these specific subgroups, highlighting its potential as a targeted therapeutic intervention for these types of AIS.

The mechanisms underlying these results may involve HUK’s ability of HUK to convert kallidinogen to kinins and vasodilators, which selectively dilate arteries in ischemic regions, thereby improving blood perfusion in the penumbra and promoting neurological recovery (40). Additionally, HUK can induce angiogenesis and neurogenesis, inhibit apoptosis in ischemic areas, and promote glucose utilization post-stroke, which may further contribute to its efficacy in reducing PIS incidence (15, 41). The lack of a significant association in some subgroups, such as those with cardioembolic infarctions or those receiving intravenous thrombolysis, may be due to the distinct pathophysiological mechanisms and treatment responses in these conditions.

Another intriguing finding of this study is that elevated blood sodium levels significantly increased the risk of PIS in the cardioembolic subgroup. There are few studies on the association between blood sodium levels and the prognosis of acute ischemic stroke patients, and the specific mechanisms behind this are not yet fully understood. A study on acute ischemic stroke patients undergoing thrombolytic therapy showed that higher blood sodium levels are associated with poor prognosis in acute ischemic stroke patients, which is consistent with our findings (42). Previous studies have clearly demonstrated a significant association between high blood sodium levels and hypertension (43). This suggests that the possible mechanism behind this is that hypertension indirectly promotes the occurrence of poor outcomes in acute ischemic stroke patients with high blood sodium levels through a mediating effect. In addition, elevated blood sodium levels may be associated with underlying comorbidities such as heart failure or renal insufficiency, which are relatively common in patients with cardioembolic stroke (44). These conditions may simultaneously affect electrolyte balance and stroke prognosis, thereby indirectly leading to the observed association. Finally, elevated blood sodium levels may reflect a state of dehydration or electrolyte imbalance, which may exacerbate cerebral ischemia and lead to neurological deterioration. Dehydration can lead to increased blood viscosity and reduced cerebral perfusion, which may exacerbate ischemic injury. However, more studies are still needed in the future to verify the conclusions and pathogenesis of this experiment.

This study also found that low BMI and high APTT are important risk factors for PIS in AIS patients. Numerous previous studies have confirmed that low BMI and abnormal coagulation function are associated with poor prognosis in cardiovascular diseases. A large-scale study showed that being underweight is associated with an increased likelihood of adverse outcomes and in-hospital mortality (45). A meta-analysis including 32 studies also indicated that, in stroke patients, being underweight is associated with increased risks of death, poor functional outcomes, and stroke recurrence (46). This is consistent with our study results, which may be because low BMI is associated with malnutrition, increased stress response, increased frequency of infections, and impaired stroke recovery, as well as exacerbated catabolic activities (47, 48). One study showed that a high international normalized ratio (INR) at admission is independently associated with death or severe disability at discharge in AIS patients (49). Another prospective study also indicated that a high level of PT-INR is associated with an increased risk of all-cause mortality in patients with coronary heart disease (50). This may be because elevated APTT suggests abnormalities in the function or quantity of coagulation factors (such as factors VII, IX, XI, etc.), leading to delayed coagulation processes. In patients with acute ischemic stroke, this delay in coagulation function may weaken the body’s ability to repair vascular damage, disrupt the dynamic balance between microthrombus formation and dissolution, increase the risk of thrombus detachment and new embolus formation, and thereby promote the occurrence of PIS. However, more studies are still needed in the future to explore the specific pathophysiological mechanisms behind this.

This study had several limitations. Firstly, the retrospective cohort design of this study means that the allocation of HUK treatment was non-random. Although we have employed multivariate adjustment methods to minimize the impact of non-random allocation on the results, we acknowledge that this design may have introduced potential selection bias. Additionally, residual confounding from unmeasured variables such as medication adherence, socioeconomic status, or unrecorded clinical characteristics may persist. Moreover, certain etiologies of neurological deterioration, such as post-stroke seizures or metabolic derangements, may not have been fully excluded in our outcome classification. In non-randomized studies, patients who received HUK treatment may differ from those who did not in terms of unmeasured or unrecorded characteristics, which could influence the interpretation of the results. For example, patients who received HUK treatment might have had differences in disease severity, treatment adherence, or comorbidity management compared to the control group. Such selection bias may affect the comparison of PIS incidence rates and thus limit the causal inference of the study results. Future studies should consider a randomized controlled trial (RCT) design to more accurately assess the effects of HUK treatment and reduce the impact of selection bias. Second, the study was conducted at a single center, which may limit the generalizability of the findings to other settings or populations. Patient demographics and clinical practices at this center may differ from those at other institutions, affecting the applicability of the results. Multi-center studies involving diverse populations are necessary to validate these findings across different healthcare environments. Third, the sample sizes for some subgroup analyses were relatively small, which may have affected the robustness and statistical power of the results. The relatively low number of cardioembolic stroke cases in this study may be due to the fact that cardioembolic stroke is primarily associated with cardiac conditions such as atrial fibrillation and myocardial infarction. Patients with these conditions may have already received specialized antithrombotic treatment in cardiology departments, or they may have preferred to seek treatment in other specialized cardiac hospitals at the time of onset. Smaller sample sizes increase the risk of type II errors, where the true effects may not be detected. Future studies should include larger patient cohorts and subgroup analyses to enhance the reliability of our conclusions. Fourth, the observational nature of the study precludes the establishment of a causal relationship between HUK treatment and reduced incidence of PIS. Although the associations observed were compelling, they could not definitively prove causality. Randomized controlled trials are needed to establish causative links and determine the efficacy of HUK treatment. Additionally, potential confounding factors such as variations in treatment protocols, patient adherence, and the presence of comorbid conditions were not fully controlled. These factors could influence outcomes and should be carefully monitored and adjusted for in future studies. Lastly, due to data limitations, key variables such as glycemic and blood pressure control during hospitalization and the timing of HUK initiation were not included in the analysis. These factors may have a significant impact on the occurrence of PIS and the effectiveness of HUK treatment. For example, poor glycemic control and uncontrolled blood pressure could exacerbate ischemic injury and influence the progression of stroke. Similarly, the timing of HUK initiation might affect its therapeutic efficacy. Future studies should collect and analyze these variables to provide a more comprehensive understanding of the factors influencing PIS and HUK treatment outcomes.

## 5 Conclusion

This study provides robust evidence that HUK significantly reduces the incidence of PIS in patients with AIS with concurrent hypertension and diabetes. Our findings indicate that HUK treatment is particularly effective in patients with large-artery atherosclerosis, small-artery occlusion, those not undergoing intravenous thrombolysis, and those with anterior circulation infarction. This treatment provides a promising strategy for reducing the risk of PIS development in patients with AIS, reducing the incidence of disability in patients with AIS, and improving the likelihood of regaining functional independence after stroke. These findings make it possible for physicians to target patients with AIS with specific risk profiles to optimize stroke management and reduce PIS incidence. Reducing the incidence of PIS in patients with AIS has broader societal benefits, including decreased healthcare costs associated with long-term care and rehabilitation of stroke survivors. Improved recovery rates and reduced disability can reduce the economic burden on healthcare systems and families.

## Data Availability

The raw data supporting the conclusions of this article will be made available by the authors, without undue reservation.
