# Supplementary material for: Role of human urinary kallikrein in reducing progressive ischemic stroke among acute ischemic stroke patients with concurrent hypertension and diabetes: a hospital-based retrospective cohort study
Source: Front Neurol. 2025 Jun 10;16:1520309. doi: 10.3389/fneur.2025.1520309 (PMC12188219; doi:10.3389/fneur.2025.1520309)
Supplement: Supplementary file 1 [file Data_Sheet_1.docx]

Supplementary Table 1. Results of the univariate analysis of general characteristics between the PIS and non-PIS groups

| Characteristics | PIS  (n=139) | Non-PIS  (n=776) | P |
| --- | --- | --- | --- |
| Gender, n (%) |  |  | 0.065^+^ |
| Male | 78 (56.1) | 499 (64.3) |  |
| Age, means±SD | 63.20±10.11 | 63.56±8.80 | 0.664 |
| BMI, means±SD | 25.20±3.45 | 25.80±3.51 | 0.071^+^ |
| History of previous diseases, n (%): |  |  |  |
| Cerebral infarction | 49 (35.3) | 303 (39.0) | 0.397 |
| Cerebral hemorrhage | 10 (7.2) | 34 (4.4) | 0.153 |
| Coronary artery disease | 28 (20.1) | 108 (13.9) | 0.057^+^ |
| AF and Arrhythmia | 7 (5.0) | 28 (3.6) | 0.419 |
| History of medicine using, n (%): |  |  |  |
| Anticoagulants | 3 (2.2) | 6 (0.8) | 0.290 |
| Antiplatelet drugs | 6 (4.3) | 30 (3.9) | 0.804 |
| Smoking, n (%) | 46 (33.1) | 224 (28.9) | 0.314 |
| Drinking alcohol, n (%) | 22 (15.8) | 175 (22.6) | 0.076^+^ |
| Stroke location, n (%): |  |  | ＜0.001^***^ |
| Anterior circulation | 97 (70.8) | 401 (52.6) |  |
| Posterior circulation | 31 (22.6) | 316 (41.4) |  |
| Anterior+posterior circulation | 9 (6.6) | 46 (6.0) |  |
| Intravenous thrombolysis | 16 (20.0) | 64 (80.0) | 0.210 |
| TOAST, n (%): |  |  | 0.469 |
| Atherosclerotic | 67 (49.3) | 355 (46.8) |  |
| Cardiogenic embolic | 6 (4.4) | 23 (3.0) |  |
| Small artery occlusion | 51 (37.5) | 278 (36.6) |  |
| Other etiologic determinants | 1 (0.7) | 3 (0.4) |  |
| Unexplained | 11 (8.1) | 100 (13.2) |  |
| HUK, n (%) | 54 (38.8) | 492 (63.4) | ＜0.001^***^ |
| Baseline NIHSS, means±SD | 3.68 (2.18) | 3.13 (2.26) | 0.009** |

^+^P＜0.1, ^*^ P < 0.05, ^**^ P < 0.01, ^***^ P < 0.001

P-values were calculated using independent sample t-tests for continuous variables and chi-squared tests for categorical variables.

Supplementary Table 2. Results of the univariate of laboratory data between PIS and non-PIS groups^#^

| Characteristics | PIS (n=139) | Non-PIS (n=776) | P Value |
| --- | --- | --- | --- |
| WBC, 10^9^／L | 8.10±2.49 | 7.96±2.42 | 0.549 |
| RBC, 10^12^ /L | 5.85±12.17 | 4.95±4.97 | 0.397 |
| PLT, 10^9^／L | 255.73±293.32 | 228.10±64.42 | 0.278 |
| PT, s  PTINR | 11.39±1.07  1.00±0.11 | 11.30±0.94  1.50±7.55 | 0.353  0.444 |
| APTT, s | 27.26±4.46 | 27.03±3.38 | 0.479 |
| FIB, g/L | 3.51±1.35 | 3.42±1.61 | 0.524 |
| TT, s | 16.67±2.22 | 16.52±3.81 | 0.669 |
| DDP, **mg/L** | 0.77±1.12 | 0.70±1.77 | 0.691 |
| BUN, mmol/L | 6.59±2.35 | 6.66±2.67 | 0.779 |
| CR, μmol/L | 78.43±66.49 | 77.15±41.96 | 0.767 |
| UA, μmol/L | 342.76±101.30 | 346.35±177.79 | 0.818 |
| TNI, pg/mL | 14.45±42.77 | 11.84±38.88 | 0.474 |
| CKMB, U/L | 1.16±1.08 | 1.59±8.39 | 0.547 |
| FBG, mmol/L | 9.20±3.48 | 8.76±3.42 | 0.170 |
| TC, mmol/L | 4.99±1.34 | 4.75±1.29 | 0.057^+^ |
| TG, mmol/L | 2.34±1.76 | 2.19±1.48 | 0.282 |
| ApoA, g/L | 1.17±0.27 | 1.19±0.42 | 0.558 |
| ApoB, g/L | 1.05±0.31 | 1.00±0.31 | 0.055^+^ |
| HDL, mmol/L | 1.05±0.29 | 1.05±0.29 | 0.951 |
| LDL, mmol/L | 3.62±6.15 | 2.93±0.99 | 0.199 |
| HCRP, mg/L | 5.43±5.26 | 5.28±10.07 | 0.933 |
| ApoA /ApoB | 1.22±0.51 | 1.31±0.56 | 0.089^+^ |
| Lpa, mg/L | 240.80±286.59 | 185.96±256.23 | 0.024^*^ |
| HCY, μmol/L | 15.46±9.09 | 14.62±10.02 | 0.406 |
| ALT, U/L | 18.90±11.38 | 20.22±14.61 | 0.321 |
| AST, U/L  albumin, g/L | 17.64±8.06  39.31±4.59 | 18.72±11.34  39.36±4.19 | 0.290  0.897 |
| globulin, g/L | 26.83±4.94 | 26.53±8.19 | 0.679 |
| GGT, U/L | 44.10±43.36 | 44.66±65.35 | 0.923 |
| alkaline phosphatase, U/L | 83.24±32.12 | 80.56±26.68 | 0.300 |
| Blood potassium, mmol/L | 5.03±11.40 | 4.38±6.97 | 0.369 |
| Blood sodium, mmol/L | 138.21±12.04 | 141.73±57.17 | 0.473 |

^#^ presented as means±SD.

^+^P＜0.1, ^*^ P < 0.05, ^**^ P < 0.01, ^***^ P < 0.001

P-values were calculated using independent sample t-tests for continuous variables and chi-squared tests for categorical variables.

Supplementary Table 3. Results of the multivariate analysis between PIS and non-PIS groups in subgroups.

| Characteristics | References | OR (95%CI) | P-value |
| --- | --- | --- | --- |
| **Atherosclerotic type:** |  |  |  |
| BMI |  | 0.91 (0.83, 0.99) | 0.034^*^ |
| Stroke location | Anterior+Posterior circulation |  |  |
| Anterior circulation lesions |  | 1.22 (0.42, 3.52) | 0.711 |
| Posterior circulation stroke |  | 0.35 (0.11, 1.13) | 0.078^+^ |
| HUK |  | 0.34 (0.19, 0.62) | <0.001^***^ |
| APTT |  | 1.11 (1.02, 1.22) | 0.020* |
| **Cardiogenic embolic type:** |  |  |  |
| Blood sodium |  | 2.22 (1.05, 4.67) | 0.037^*^ |
| WBC |  | 1.35 (0.83, 2.17) | 0.225 |
| **Small artery occlusion type:** |  |  |  |
| Male |  | 1.30 (0.59, 2.86) | 0.510 |
| Drinking alcohol |  | 0.52 (0.19, 1.47) | 0.220 |
| Stroke location | Anterior+Posterior circulation |  |  |
| Anterior circulation lesions |  | - |  |
| Posterior circulation stroke |  | - |  |
| HUK |  | 0.33 (0.17, 0.66) | 0.002^**^ |
| CR |  | 0.98 (0.96, 1.00) | 0.034 |
| HDL |  | 2.00 (0.60, 6.63) | 0.257 |
| Globulin |  | 1.06 (0.97, 1.15) | 0.207 |
| **Unexplained type:** |  |  |  |
| HUK |  | 0.11 (0.001, 13.02) | 0.363 |
| APTT |  | 1.08 (0.66, 1.77) | 0.750 |
| HCRP |  | 1.38 (0.95, 2.01) | 0.091^+^ |
| HCY |  | 0.96 (0.63, 1.47) | 0.846 |
| **Intravenous thrombolysis:** |  |  |  |
| Smoking |  | 4.75 (1.37, 16.46) | 0.014^*^ |
| Admission temperature |  | 68.19 (1.68, 2769.01) | 0.025^*^ |
| **Non-Intravenous thrombolysis:** |  |  |  |
| Stroke location | Anterior+Posterior circulation |  |  |
| Anterior circulation lesions |  | 1.46 (0.65, 3.27) | 0.362 |
| Posterior circulation stroke |  | 0.53 (0.22, 1.27) | 0.156 |
| HUK |  | 0.33 (0.22, 0.50) | <0.001^***^ |
| **Anterior circulation lesions:** |  |  |  |
| BMI |  | 0.89 (0.83,0.95) | 0.001** |
| HUK |  | 0.20 (0.12, 0.34) | <0.001^***^ |
| TT |  | 1.08 (0.94,1.23) | 0.268 |
| CR |  | 0.99 (0.98,1.00) | 0.047* |
| ApoB |  | 2.54 (1.16,5.58) | 0.020* |
| Lpa |  | 1.00 (1.00,1.00) | 0.032* |
| **Posterior circulation stroke:** |  |  |  |
| Coronary artery disease |  | 2.61 (1.11, 6.17) | 0.028^*^ |
| History of antiplate drugs |  | 5.29 (1.46, 19.19) | 0.011^*^ |
| **Anterior+Posterior circulation:** |  |  |  |
| FIB |  | 1.90 (0.84, 4.30) | 0.126 |
| TG |  | 2.13 (1.02, 4.44) | 0.043^*^ |
| Albumin |  | 0.78 (0.58, 1.05) | 0.097^+^ |

^+^P＜0.1, ^*^P < 0.05, ^**^ P < 0.01, ^***^ P < 0.001

The binary logistic regression model was performed both unadjusted and adjusted for potential confounders, which included factors with a p-value < 0.1 in the univariate analysis. In the small vessel occlusion subgroup, due to the large disparity in the distribution of case numbers by stroke location, the results of the multivariate analysis were not reported.
